# Evaluation of Health-Related Values and Preferences of Adults Who Were Preterm Infants and Parents of Preterm Infants Concerning Use of Prophylactic Cyclooxygenase Inhibitor Drugs

**DOI:** 10.1001/jamanetworkopen.2023.2273

**Published:** 2023-03-09

**Authors:** Souvik Mitra, Tara Hatfield, Marsha Campbell-Yeo, Jon Dorling, Bradley C. Johnston

**Affiliations:** 1 Division of Neonatal-Perinatal Medicine, Department of Pediatrics, Dalhousie University, Halifax, Nova Scotia, Canada; 2 Department of Community Health and Epidemiology, Dalhousie University, Halifax, Nova Scotia, Canada; 3 Division of Neonatal-Perinatal Medicine, IWK Health, Halifax, Nova Scotia, Canada; 4 School of Nursing, Faculty of Health, Dalhousie University, Halifax, Nova Scotia, Canada; 5 Department of Neonatology, Southampton Children’s Hospital, Southampton, United Kingdom; 6 Department of Nutrition, Texas A&M University, College Station; 7 Department of Epidemiology, Texas A&M University, College Station; 8 Department of Biostatistics, Texas A&M University, College Station

## Abstract

**Importance:**

There is wide variability in the use of prophylactic cyclooxygenase inhibitor (COX-I) drugs to prevent morbidity and mortality in preterm infants. Parents of preterm infants are rarely involved in this decision-making process.

**Objective:**

To explore the health-related values and preferences of adults who were preterm infants and families of preterm infants concerning the prophylactic use of indomethacin, ibuprofen, and acetaminophen initiated within the first 24 hours after birth.

**Design, Setting, and Participants:**

This cross-sectional study used direct choice experiments conducted in 2 phases of virtual video-conferenced interviews between March 3, 2021, and February 10, 2022: (1) a pilot feasibility study and (2) a formal study of values and preferences, using a predefined convenience sample. Participants included adults born very preterm (gestational age <32 weeks) or parents of very preterm infants currently in the neonatal intensive care unit (NICU) or having graduated from the NICU in the last 5 years.

**Main Outcomes and Measures:**

Relative importance of clinical outcomes, willingness to use each of the COX-Is when presented as the only option, preference for using prophylactic hydrocortisone vs indomethacin, willingness to use any of the COX-Is when all 3 options are available, and relative importance of having family values and preferences included in decision-making.

**Results:**

Of 44 participants enrolled, 40 were included in the formal study (31 parents and 9 adults born preterm). The median gestational age of the participant or the participant’s child at birth was 26.0 (IQR, 25.0-28.8) weeks. Death (median score, 100 [IQR, 100-100]) and severe intraventricular hemorrhage (IVH) (median score, 90.0 [IQR, 80.0-100]) were rated as the 2 most critical outcomes. Based on direct choice experiments, most participants were willing to consider prophylactic indomethacin (36 [90.0%]) or ibuprofen (34 [85.0%]), but not acetaminophen (4 [10.0%]) when offered as the only option. Among participants who initially chose indomethacin (n = 36), if prophylactic hydrocortisone was offered as a potential therapy with the caveat that both cannot be used simultaneously, only 12 of 36 (33.3%) preferred to remain with indomethacin. Variability in preference was noted when all 3 COX-I options were available, indomethacin (19 [47.5%]) being the most preferred option followed by ibuprofen (16 [40.0%]), while the remainder opted for no prophylaxis (5 [12.5%]).

**Conclusions and Relevance:**

The findings of this cross-sectional study of former preterm infants and parents of preterm infants suggest that there was minimal variability in how participants valued the main outcomes, with death and severe IVH being rated as the 2 most important undesirable outcomes. While indomethacin was the most preferred form of prophylaxis, variability was noted in the choice of COX-I interventions when participants were presented with the benefits and harms of each drug.

## Key Points

**Question:**

What are the values and preferences of parents of preterm infants and adults who had been born preterm concerning the use of cyclooxygenase inhibitor (COX-I) prophylaxis to prevent morbidity and mortality?

**Findings:**

In this cross-sectional study that enrolled 44 participants, death and severe intraventricular hemorrhage were rated as the 2 most critical outcomes. Among 40 participants, the preference for COX-I prophylaxis was variable, with indomethacin (19 [48%]) being the most preferred option, followed by ibuprofen (16 [40%]), while the remainder opted for no prophylaxis (5 [13%]).

**Meaning:**

These findings suggest that death was the most undesirable outcome and indomethacin was the most preferred prophylactic COX-I to use in preterm infants.

## Teaser

This cross-sectional study explores the health-related values and preferences of adults who had been preterm infants and parents of preterm infants concerning the use of cyclo-oxygenase inhibitors in preterm infants to prevent patent ductus arteriosus–related morbidity and mortality.

## Introduction

Infants born extremely preterm (gestational age ≤28 weeks) are at a high risk of complications such as severe intraventricular hemorrhage (IVH), necrotizing enterocolitis (NEC), and chronic lung disease (CLD). A common contributor for these pathophysiological mechanisms is postulated to be the patent ductus arteriosus (PDA).^1^ Currently available pharmacotherapeutic options to prevent PDA and related complications include cyclooxygenase inhibitors (COX-Is) such as indomethacin, ibuprofen, and acetaminophen. COX-Is themselves are associated with serious adverse effects such as NEC and gastrointestinal tract perforation.^2,3^ Recent availability of prophylactic hydrocortisone as a potential effective option to prevent death or CLD also presents a dilemma to clinicians, as concomitant use of prophylactic indomethacin and hydrocortisone significantly increases the risk of gastrointestinal tract perforation.^4^ Given the potential risks of COX-I use, there exists wide variation in clinical practice regarding COX-I prophylaxis in preterm infants.^3,5^ The decision on COX-I pharmacoprophylaxis is likely driven by the perceived benefits vs potential risks as determined by the treating physician, with little input from families regarding their values and preferences.

Health-related values refer to the perspectives, beliefs, expectations, and goals for health and life of the parents and guardians for their infants, while preferences refer to the processes that families use in considering the potential benefits, harms, costs, and inconveniences of the management options in relation to one another.^6^ Consequently, it is plausible that the preference for or against an intervention is determined by the relative importance of the health outcomes that the family attaches to available management strategies.^6,7,8^ COX-I prophylaxis involves such a trade-off between long-term benefits and serious short-term adverse effects. Therefore, it is imperative that family preferences are included in clinical guidelines for COX-I prophylaxis in preterm infants.

There is a dearth of research on values and preferences of families and former preterm infants in this context, with no evidence from previous guidelines that the explicit values and preferences of families have been incorporated. The objective of this study was to explore the health-related values and preferences of former preterm infants and families of preterm infants on the use of COX-I prophylaxis for preventing PDA-related morbidity and mortality.

## Methods

### Study Design and Population

This cross-sectional study followed the Strengthening the Reporting of Observational Studies in Epidemiology (STROBE) reporting guideline. Adults born very preterm (gestational age <32 weeks) or families of very preterm infants currently in the neonatal intensive care unit (NICU) or having graduated from the NICU in the last 5 years were included from across Canada and the United Kingdom. The study was approved by the IWK Health Research Ethics Board, and all participants provided electronically signed written informed consent.

The study was planned in 2 phases. Phase 1, a pilot feasibility study, aimed to test our study questionnaire and provided an opportunity to modify any logistic or methodological issues. Phase 2, a formal study of values and preferences, used our pretested interview questionnaire to describe the variability in health-related values and preferences of former preterm infants and families concerning prophylactic use of COX-Is.

### Recruitment Strategy

A convenience sampling strategy was used with emphasis on recruitment of underrepresented groups such as Black and Indigenous populations as well as participants with a low level of educational attainment to obtain a representative sample of patients and families in the NICU. Participants were contacted while their infants were admitted to the IWK Health NICU, through the IWK Health perinatal follow-up clinic, and through representatives of local and national Canadian Premature Babies Foundation parent partner organizations. Informed consent was obtained from each participant prior to their respective interviews. Participants with limited understanding of English were excluded. The study was conducted virtually using recorded video-conference interviews on the Zoom platform (Zoom Video Communications).

### Study Procedures

A structured survey and semistructured interview script were developed by the research team (eMethods and eFigure in Supplement 1). The interview consisted of the following components:

1. Baseline demographic questionnaire: The questionnaire included type of participant, age range, highest educational level attained, race and ethnicity (self-reported), country of origin, and gestational age of the participant or the participant’s child at birth.
2. Standardized description of health states: In this section, the participants were provided information on PDA-related complications and available preventive options (eMethods in Supplement 1).
3. Eliciting importance of outcomes: A numeric rating scale was used to elicit the perceived importance of the following outcomes: death, severe IVH, NEC, and CLD on a scale of 0 to 100, with 0 being least important and 100 being the most critical outcome. Patent ductus arteriosus was also included in the numeric rating scale, though it was not identified as a critical outcome, as the standardized descriptions of health states included PDA in addition to severe IVH, NEC, and CLD.
4. Direct-choice elicitation for treatment preferences: A direct-choice experimental design was used to assess the proportion of participants willing to accept prophylactic use of any of the 3 COX-Is.^9,10^ Evidence on benefits and harms for each of the 3 medications were presented for the outcomes of death, severe IVH, NEC, and CLD using a visual decision aid created from the MAGICapp software^11^ (eFigure in Supplement 1). The decision aid was accompanied by the baseline risk and absolute risk reduction for each outcome, followed by the overall certainty of evidence for each outcome as determined using the GRADE (Grading of Recommendations Assessment, Development and Evaluation) methodology.^12^ Participants were then asked to choose yes or no for each pharmacotherapeutic option. Those participants who chose indomethacin were further presented with the benefits and harms of prophylactic hydrocortisone, with the caveat that both cannot be used together, to explore their choice when presented with the option of choosing between indomethacin and hydrocortisone.
5. Semistructured interview on determinants of treatment preferences: To explore the determinants and any emerging themes that affected how and why participants chose certain treatment preferences, participants were asked to list the most important factors behind their choice of therapy.
6. Relative importance of having family values and preferences included in decision-making: Given the risk of information overload in the first 24 hours, participants were asked how important it was for them to have their values and preferences included in decision-making for use of prophylactic COX-Is. They were asked to choose 1 of the 4 options provided (not important, somewhat important, important, or very important) with a brief description of the implications of each choice.

For the pilot study, we used data on use of prophylactic indomethacin, ibuprofen, and acetaminophen in preterm infants available from existing evidence published in the *Cochrane Database of Systematic Reviews*.^13,14,15^ For the formal study, updated evidence from the recent Cochrane review and network meta-analysis by Mitra et al^16^ was used. Evidence on prophylactic use of hydrocortisone was drawn from a 2019 individual patient data meta-analysis by Shaffer et al.^17^

### Outcomes

The outcome measures included (1) relative importance of clinical outcomes; (2) willingness to use each COX-I when presented as the only option; (3) preference for using prophylactic hydrocortisone vs indomethacin; (4) willingness to use any of the COX-Is when all 3 options are available; and (5) relative importance of having family values and preferences included in decision-making. The qualitative component of the interview attempted to identify themes related to the choice of prophylaxis based on participants’ perceptions of the therapeutic value of each COX-I.

### Statistical Analysis

#### Quantitative Analysis

Categorical data are expressed as frequencies and percentages. Continuous data are expressed as mean (SD) for parametric data and median (IQR) for nonparametric data. Post hoc exploratory analyses by participant group (parent of preterm infant vs adult born preterm) were conducted using the Mann-Whitney test, *z* test, χ^2^ test, or Fisher exact test as applicable. Statistical inferences were based on 2-tailed tests with statistical significance set at *P* < .05. All quantitative analyses were conducted using Minitab software, version 19 (Minitab LLC).

#### Qualitative Analysis

The virtual interviews were recorded and transcribed verbatim for qualitative analysis using a thematic analysis approach.^18,19^ Transcripts were coded and the codes were sorted into themes. One researcher (S.M.) conducted all interviews, coded transcripts, and sorted relevant sections of the transcript into major themes using the NVivo software, version 12 (QSR International). A second research coordinator (T.H.) who was not involved in any of the interviews independently coded a randomly selected sample of 20 transcripts to validate the work. Validity of the original coding was established if no additional themes were identified. Coding frequency of the emerging major themes was presented as percentages.

## Results

Forty-four participants were enrolled during the study period between March 3, 2021, and February 10, 2022. The study population was predominantly White (35 [80.0%]) with a much smaller representation of Black individuals (1 [2.3%]), Indigenous peoples of Canada (2 [4.5%]), and individuals of other race or ethnicity (including Arab, Chinese, and South Asian) (6 [13.6%]).

### Pilot Phase 1 Study

Seven participants were enrolled during the phase 1 pilot (5 parents and 2 adults born preterm) (eTable 1 in Supplement 1) between March 3 and May 11, 2021. The median age of participants or their children at birth was 24.0 (IQR, 23.0-28.0) months; all 7 participants were White. Based on the feedback of the participants, the following changes were made to the formal phase 2 study:

1. Sex was removed from the demographic questionnaire, as both parents often participated together in the interviews.
2. In the pilot study, participants were asked to rate 5 clinical outcomes (severe IVH, severe developmental delay, CLD, NEC, and gastrointestinal tract perforation) on a numeric rating scale of 0 to 100, assuming 100 was the worst possible state of health, while 0 was the best possible state of health. These outcomes were chosen based on consensus of the study team and input from parent partners from the Canadian Premature Babies’ Foundation, because these were perceived as the most important clinical outcomes related to use of prophylactic COX-I. Death was initially not included in these 5 outcomes as it was assumed to be the worst possible state of health. The participants believed that for some parents, death may not always be the worst possible state of health. Therefore, death was added as one of the clinical outcomes to be rated on the numeric rating scale. The participants furthermore believed that evidence on too many outcomes was presented. The unanimous consensus from all 7 participants was to choose death, severe IVH, NEC, and CLD as the 4 outcomes to be presented in the direct choice experiments in the phase 2 study.
3. Based on the recruitment rate in the pilot phase (approximately 3-4 participants per month), a convenience sample target of 40 was determined, anticipating that 40 participants would allow for study completion by Spring of 2022.

### Formal Phase 2 Study

Forty participants were recruited in the formal phase 2 study between October 5, 2021, and February 10, 2022, including 3 participants (2 parents and 1 adult) who had also participated in the pilot phase. Of the 40 participants, 31 (77.5%) were parents of infants born very preterm, while 9 (22.5%) were adults who were born very preterm. The overall median gestational age of the participants or their children at birth was 26.0 (IQR, 25.0-28.8) weeks. Most participants were White (31 [77.5%]), with 1 (2.5%) Black, 2 (5.0%) Indigenous, and 6 (15.0) other. Twenty-seven participants (67.5%) had a university degree. The demographic profile of the participants in the formal phase 2 study is presented in Table 1.

**Table 1.** Demographic Profile of Participants in the Formal Phase 2 Study

Characteristic	Values (N = 40)^a^
Type of participant	
Parent of a very preterm infant	31 (77.5)
Adult former preterm infant	9 (22.5)
Age, y	
18-24	7 (17.5)
25-34	20 (50.0)
35-44	12 (30.0)
45-54	1 (2.5)
Race and ethnicity	
Black	1 (2.5)
Indigenous peoples of Canada	2 (5.0)
White	31 (77.5)
Other^b^	6 (15.0)
Highest level of education completed	
Less than high school	0
High school	5 (12.5)
College or trade school certificate or diploma	8 (20.0)
University undergraduate degree	16 (40.0)
University postgraduate degree	11 (27.5)
Country of origin	
Canada	35 (87.5)
United Kingdom	3 (7.5)
Other	2 (5.0)
Gestational age of the participant or participant’s child at birth, median (IQR), wk	26.0 (25.0-28.8)

^a^ Unless indicated otherwise, data are expressed as No. (%) of participants.

^b^ Includes Arab, Chinese, and South Asian.

#### Rating of Importance of Outcomes

On the numeric rating scale, death was rated as the most serious outcome (median score, 100 [IQR, 100-100]). Severe IVH followed as the next most serious outcome (median score, 90.0 [IQR, 80.0-100]) (Table 2).

**Table 2.** Value Placed on Outcomes

Outcome	Score, median (IQR)^a^
Death	100 (100-100)
Severe intraventricular hemorrhage	90.0 (80.0-100)
Chronic lung disease	70.0 (60.0-80.0)
Necrotizing enterocolitis	80.0 (70.0-90.0)
Patent ductus arteriosus	75.0 (52.5-90.0)

^a^ Scores range from 0 to 100, 0 being least important and 100 being the most critical outcome.

#### Direct Choice Elicitation of Treatment Preferences and Rationale for Choices

When offered as the only available option, most participants chose indomethacin (36 [90.0%]) and ibuprofen (34 [85.0%]), while only a small proportion chose acetaminophen (4 [10.0%]) (Table 3). Among participants who initially chose indomethacin (n = 36), when prophylactic hydrocortisone was offered as a potential therapy with the caveat that both cannot be used simultaneously, 12 of 36 (33.3%) still preferred indomethacin (Table 4).

**Table 3.** Preference for Prophylactic Therapies Among All Participants

Drug	Participants who chose drug, No. (%) (n = 40)	Thematic analysis summary	
		Major themes	Participants who chose and alluded to this theme, No. (%)
**When therapies are offered as the only option (vs no prophylaxis)**			
Indomethacin	36 (90.0)	Reduces death (critical outcome)	22 (61.1)
		Reduces severe IVH (critical outcome)	21 (58.3)
		Possible increase in CLD less worrisome	12 (33.3)
		Higher certainty in evidence for benefit (reduction in death, severe IVH, NEC), lower certainty in evidence for harm (increase in CLD)	9 (25.0)
Ibuprofen	34 (85.0)	Reduces death (critical outcome)	14 (41.2)
		Reduces severe IVH (critical outcome)	15 (44.1)
		No obvious evidence of harm	10 (29.4)
Acetaminophen	4 (10.0)	Not enough evidence, high uncertainty^a^	25 (69.4)
		Possible harm with increased risk of IVH^a^	9 (25.0)
**When all 3 options are available (vs not choosing anything)**			
Indomethacin	19 (47.5)	Overall certainty of benefit better with indomethacin	13 (68.4)
Ibuprofen	16 (40.0)	No overall harm	8 (50.0)
		Indomethacin definitely cannot be used with hydrocortisone, hence going with the second best option	7 (43.8)
No prophylaxis	5 (12.5)	Would want to give hydrocortisone if offered	2 (40.0)

Abbreviations: CLD, chronic lung disease; IVH, intraventricular hemorrhage; NEC, necrotizing enterocolitis.

^a^ The major themes reflect the rationale of participants for not choosing acetaminophen (n = 36).

**Table 4.** Preference for Indomethacin vs Hydrocortisone Prophylaxis Among Participants Who Initially Opted for Indomethacin

Drug	Participants who chose drug, No. (%) (n = 36)	Thematic analysis summary	
		Major themes	Participants who chose and alluded to this theme, No. (%)
Indomethacin	12 (33.3)	Reduction of IVH is important; also reduces death	8 (66.7)
Hydrocortisone	24 (66.7)	Survival and survival without CLD better with hydrocortisone compared with indomethacin	18 (75.0)

Abbreviations: CLD, chronic lung disease; IVH, intraventricular hemorrhage.

Thematic analysis showed that for indomethacin, reduction in death and severe IVH with moderate certainty was the primary driver for the participants’ choice in favor (Table 3). However, when prophylactic hydrocortisone was offered to those who chose indomethacin, two-thirds of participants indicated that they would prefer hydrocortisone over indomethacin, as hydrocortisone offered improved survival over indomethacin (Table 4). Similar to indomethacin, the primary motivation behind choosing ibuprofen over no treatment was possible reduction in the critical outcomes of death and severe IVH. Most participants opted against acetaminophen as they believed that the evidence was highly uncertain (Table 3).

When all 3 COX-I options were available, 19 of 40 participants (47.5%) chose indomethacin, 16 (40.0%) chose ibuprofen, and 5 (12.5%) opted for no COX-I prophylaxis. Thematic analysis showed that those who chose indomethacin believed that the overall certainty for benefit was better with indomethacin; those who chose ibuprofen indicated that there seemed to be no overall harm and in addition they would like to keep the option of using prophylactic hydrocortisone open, which was not possible with indomethacin. For the remaining participants who opted for no prophylaxis, the primary motivation was preference for hydrocortisone (Table 3).

#### Relative Importance of Having Family Values and Preferences Included in the Decision-making

Most participants believed that it was somewhat important (22 [55.0%]) or important (14 [35.0%]) to be informed of the benefits and harms of the pharmacoprophylactic options prior to making a clinical decision to use the drug or refrain from use (Table 5). Those who indicated that it was somewhat important believed that the first 24 hours after birth was overwhelming. Therefore, though they would like to be informed about the benefits and harms of the therapies, they would trust the clinician’s judgment. Those who indicated that it was important needed to be actively involved in this decision-making process (Table 5).

**Table 5.** Importance of Having Participant Values and Preferences Included in Decision-making

Choice	Participants who chose option, No. (%) (n = 40)	Thematic analysis summary	
		Major themes	Participants who chose and alluded to this theme, No. (%)
Not important (I do not want to know the details; I will defer this decision to the physician)	3 (7.5)	NA	NA
Somewhat important (I would like to know the benefits and harms of treatment and the rationale behind the physician’s decision; but I will follow what the physician feels best)	22 (55.0)	First 24 h after birth is overwhelming, lot of things to process; so would want to be aware of benefits and harms, but will trust clinician’s judgment	17 (77.3)
Important (I want to have a discussion with the physician regarding the benefits and harms related to the most important outcomes and then make a decision together)	14 (35.0)	Would like to be involved in the discussion regarding benefits and harms	6 (42.9)
Highly important (I would like to make the decision myself based on the information provided)	1 (2.5)	NA	NA

Abbreviation: NA, not applicable.

#### Post Hoc Exploratory Analysis

Post-hoc exploratory analysis failed to demonstrate any statistically significant differences in the responses between parents of preterm infants vs those adults who were born very preterm. The results of this analysis are provided in eTable 2 in Supplement 1.

## Discussion

This cross-sectional study included 44 participants, 40 of whom were included in the formal phase 2 study. Our results showed that death and severe IVH were the 2 most serious outcomes that participants would consider in relation to prophylactic COX-I use in preterm infants. Most participants were willing to consider the use of prophylactic indomethacin or ibuprofen, but not acetaminophen when offered as the only option. There was some variability in the preference when all 3 COX-I options were available, with indomethacin (47.5%) being the most preferred option followed by ibuprofen (40.0%).

To our knowledge, this is the first study to explore the health-related values and preferences for use of available pharmacoprophylactic COX-Is in preterm infants. The study was developed through an iterative process of pilot testing and feedback from relevant stakeholders, including parents of preterm infants, adults born preterm, neonatal clinicians, and experts in clinical epidemiology. The information was shared using decision aids that incorporated absolute risk differences and certainty of evidence.^20,21^ It has been previously shown that families better understand absolute risk reduction and visual aids (such as icon arrays and bar graphs) for risk communication, and decision-making is likely to be improved when decision-makers have knowledge of the certainty of evidence.^22,23^

There is limited evidence on family values and preferences for neonatal interventions and outcomes. The only previous study by AlFaleh et al,^24^ which explored maternal preference for indomethacin prophylaxis vs symptomatic PDA treatment in preterm infants, showed findings similar to our study results despite distinct methodological differences. That study, conducted in Saudi Arabia, enrolled 290 participants, 75% of whom were healthy pregnant women at 23 to 28 weeks’ gestation, whereas in our study, all participants have had the experience of living through 1 or more of the health outcomes discussed in the interview. Despite the methodological differences, there were noticeable similarities in the results. In the study by AlFaleh et al,^24^ severe IVH was viewed as the most serious outcome, and participants had a strong preference for prophylactic indomethacin (82%). Similarly, in our study, severe IVH was rated as the most serious outcome after death, and 90.0% of participants preferred prophylactic indomethacin if this was available as the only option. Of note, a 2019 study on development of a core outcome set for neonatal research^25^ demonstrated that the 4 top-ranked outcomes by severity from a patient and parent perspective were death, NEC, sepsis, and brain injury on imaging. This suggests that despite limited evidence on how parents and patients value neonatal outcomes, regardless of the study type or setting, death and severe IVH are highly valued with respect to their seriousness.

### Limitations

There are several limitations that should be considered while interpreting the results of the study. First, our sample size for the formal phase 2 study was limited to 40 participants and the study population was predominantly White (77.5%) with a much smaller representation of Black (2.5%) or Indigenous (5.0%) populations. Further, most participants (67.5%) had a university degree. As a result, our sample size was insufficient to explore potential differences in responses based on race and ethnicity, educational attainment, geographic region, or health care system. The primary rationale for limiting the sample size to 40 participants was to ensure timely study completion so that evidence from this study and the corresponding systematic review^16^ remained relevant and up-to-date for a guideline development exercise on this topic using the GRADE Evidence-to-Decision framework planned by members of the authoring team for Spring 2022.^26^ Additional large studies are required to explore whether participant preferences and their rationale for prophylactic interventions vary based on race and ethnicity, educational attainment, and socioeconomic backgrounds. Second, all interviews were conducted by a single individual, which increases the risk of implicit bias during the interview process, despite having a structured interview format, which in turn may influence participant responses. However, independent thematic analysis by a second researcher of 20 participants failed to identify any additional themes from the interview transcripts. Third, participant preferences for or against an intervention may be directly related to the evidence presented. In this study, we chose to present evidence on clinically meaningful outcomes obtained from randomized clinical trials only as they are deemed to be the most unbiased source of evidence. Additional data on adverse events such as gastrointestinal tract perforation obtained from observational studies may have resulted in more conservative responses with more parents refraining from prophylactic COX-I therapy.

## Conclusions

In this cross-sectional study, death and severe IVH were rated as the 2 most important undesirable outcomes in relation to prophylactic COX-I use in preterm infants. While indomethacin was the most preferred form of prophylaxis, variability was noted in the choice of COX-I interventions when participants were presented with the benefits and harms of each drug. This study offers unique insights into how parents of preterm infants and adults who were preterm infants value clinical outcomes and perceive the benefits and harms of prophylactic interventions for preventing morbidity and mortality. The knowledge of parent and patient preferences for COX-I pharmacoprophylaxis generated from this study should inform guideline developers as they formulate guideline recommendations on this topic. Further, if recommended by clinical guidelines, the decision aids used in this study may be used for shared decision-making with families. This and similar studies on family preferences may therefore act as a novel bridge for translating the evidence generated through a systematic review of evidence into clinical practice guideline recommendations.

## Notes

Supplement 1. **eMethods.** Description of Health Conditions **eFigure.** Structured Interview Slides **eTable 1.** Demographic Profile of Participants in the Pilot Phase 1 Study (n = 7) **eTable 2.** Post hoc Exploratory Analysis: Responses by Participant Group

Supplement 2.
Data Sharing Statement
