# Supplementary material for: Evaluation of Health-Related Values and Preferences of Adults Who Were Preterm Infants and Parents of Preterm Infants Concerning Use of Prophylactic Cyclooxygenase Inhibitor Drugs
Source: JAMA Netw Open. 2023 Mar 9;6(3):e232273. doi: 10.1001/jamanetworkopen.2023.2273 (PMC9999240; doi:10.1001/jamanetworkopen.2023.2273)
Supplement: Supplement 1. — eMethods. Description of Health Conditions eFigure. Structured Interview Slides eTable 1. Demographic Profile of Participants in the Pilot Phase 1 Study (n = 7) eTable 2. Post hoc Exploratory Analysis: Responses by Participant Group [file jamanetwopen-e232273-s001.pdf]

Supplementary Online Content

Mitra S, Hatfield T, Campbell-Yeo M, Dorling J, Johnston BC. Evaluation of health-related values and preferences of adults who were preterm infants and parents of preterm infants concerning use of prophylactic cyclooxygenase inhibitor drugs. *JAMA Netw Open*. 2023;6(3):e232273. doi:10.1001/jamanetworkopen.2023.2273

eMethods. Description of Health Conditions
eFigure. Structured Interview Slides
eTable 1. Demographic Profile of Participants in the Pilot Phase 1 Study (n = 7)
eTable 2. Post hoc Exploratory Analysis: Responses by Participant Group

This supplementary material has been provided by the authors to give readers additional information about their work.

© 2023 Mitra S et al. *JAMA Network Open*.

eMethods. Description of Health Conditions

<u>Patent ductus arteriosus (PDA)</u>

Ductus arteriosus is a small passage in the heart. Normally, this passage closes shortly after birth when the baby takes their first breaths. When the ductus arteriosus remains open after birth, it is called a patent ductus arteriosus or PDA. This is a heart defect that may resolve on its own, but it can increase the risk for more serious outcomes.

<u>Potential complications of PDA</u>

The following outcomes are the most common and most concerning outcomes associated with a PDA, affecting the brain, the gut and the lungs.

The impact of these outcomes can range from being transient with minimal long-term effects to very severe long-lasting effects and can even cause death. The following descriptions include the worst-case scenarios for each of these conditions. We will describe each outcome, then ask you how you perceive and rate the seriousness of these possible outcomes

<u>Lung-related complications</u>

A premature baby's lungs are not fully developed, and babies may require breathing support with the help of a ventilator device with or without a breathing tube for extended periods. Being born early and prolonged use of breathing support may cause injury or damage to the lungs, known as chronic lung disease. A PDA can lead to extra blood flow to the lungs, which increases the need for breathing support and as a result increases the risk of lung damage and chronic lung disease.

<u>Chronic lung disease:</u>

When the lungs are damaged, some of the damaged lung tissue may be replaced by scar tissue, and the lungs may be unable to work properly for several weeks and months. Babies with CLD will have trouble breathing and may require oxygen or hospitalization for long periods of time, which may affect development. Babies with CLD often go home requiring oxygen therapy and have a

© 2023 Mitra S et al. *JAMA Network Open*.

higher chance of getting hospitalized multiple times with breathing problems, especially in the first year of life, and have a higher chance of dying compared to babies who do not have CLD.

Gut-related complications

Premature babies have underdeveloped digestive systems, and are at an increased risk of damage to their gut from many different causes. A PDA can reduce the blood flow to the gut, which increases the risk of damage.

Necrotizing enterocolitis (NEC) is a disease of the gut that primarily affects premature and medically fragile infants. In the most severe forms, large sections of the gut are damaged, becoming black and dead and may perforate. Many babies with NEC will require surgery to remove the dead and perforated bowel, followed by a prolonged course of hospital stay and intravenous nutrition. 20-30% of babies diagnosed with NEC will die in spite of medical/surgical treatment. Babies who survive following NEC may have lifelong developmental problems likely related to multiple surgeries, frequent hospitalizations and poor nutrition.

Brain-related complications

Blood vessels inside a premature baby's brain are thin and fragile. They are sensitive to changes in blood flow and they can tear easily. A PDA results in changes in normal blood flow, which increases the risk of torn blood vessels and bleeding in the brain.

Severe Intraventricular Hemorrhage (IVH):

Torn blood vessels may cause bleeding inside the ventricles of brain (ventricles are chambers inside the brain filled with fluid). This is called intraventricular hemorrhage (IVH). IVHs are graded from 1 to 4 based on their severity. When the bleeding fills up and stretches out the ventricles or involves the surrounding brain matter it is known as severe intraventricular hemorrhage (grades 3 and 4). Severe IVH increases the risk for long lasting brain damage and severe developmental delay later in life.

© 2023 Mitra S et al. *JAMA Network Open*.

eFigure. Structured Interview Slides

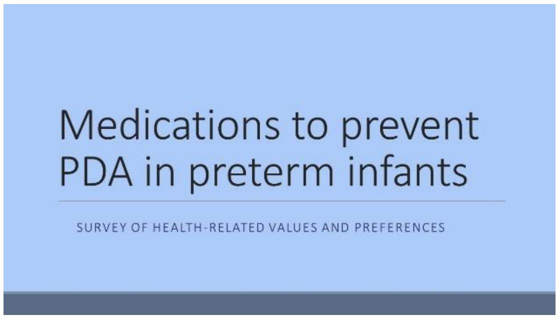

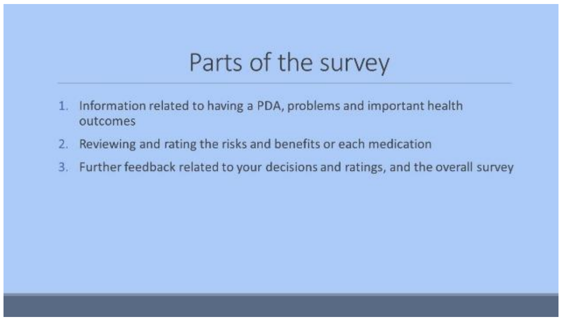

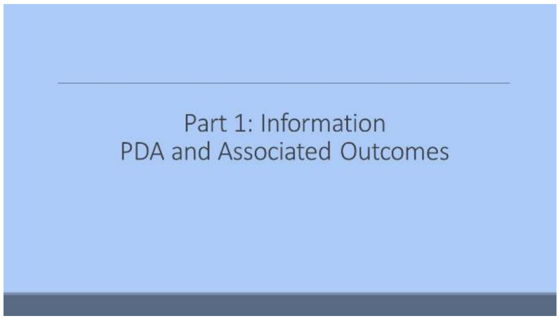

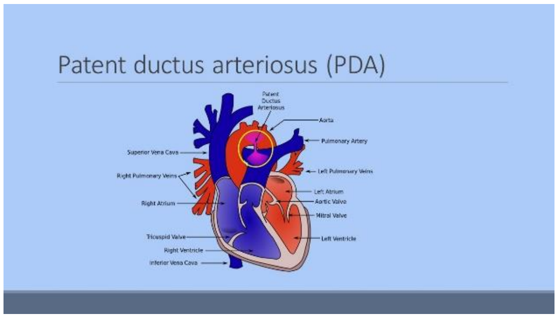

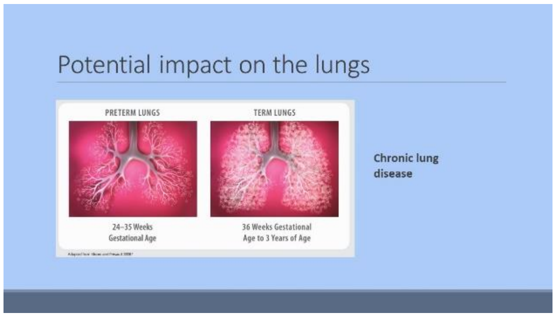

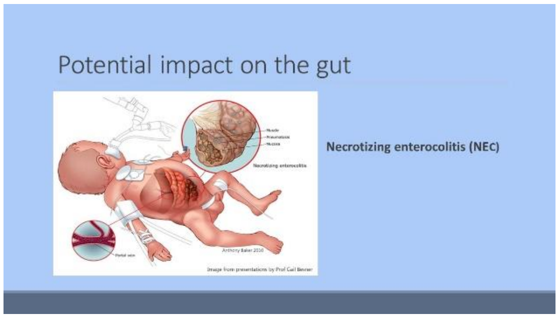

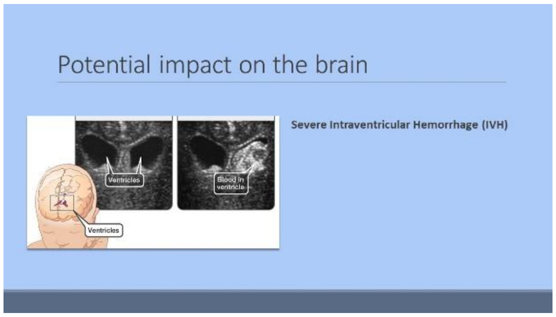

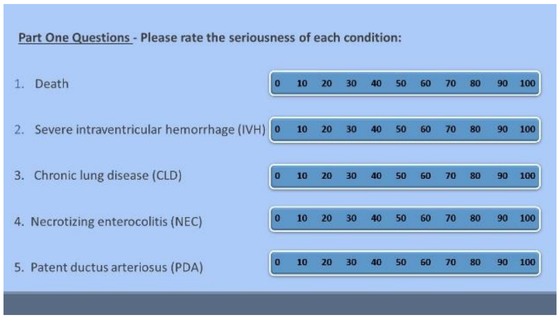

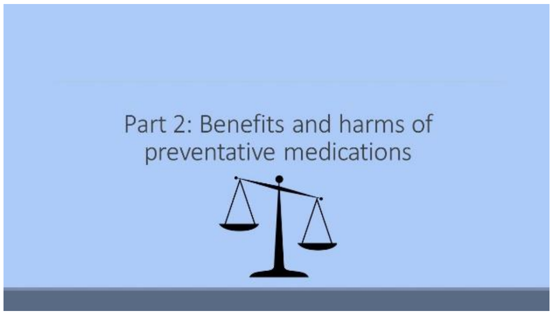

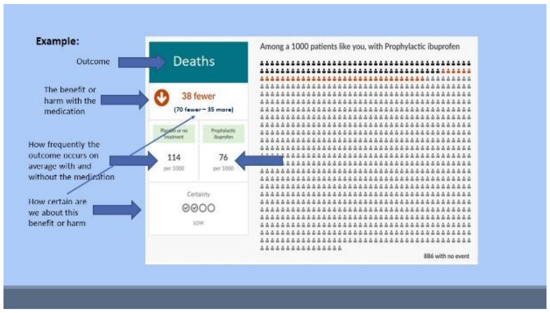

© 2023 Mitra S et al. *JAMA Network Open*.

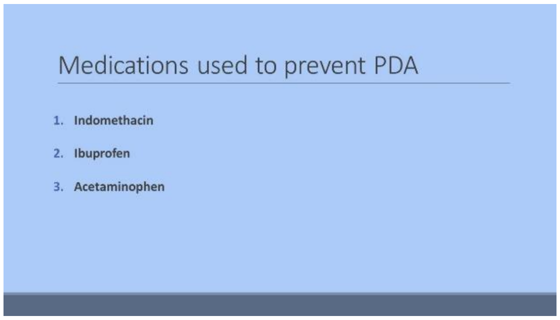

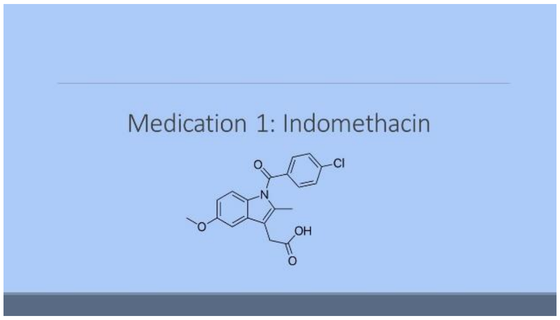

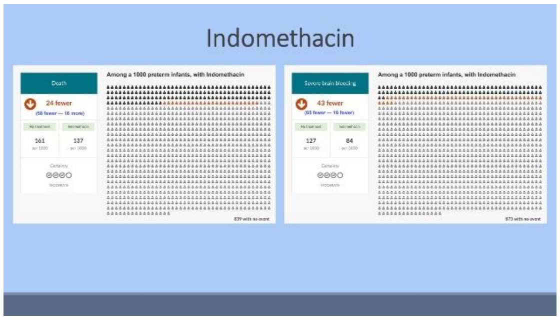

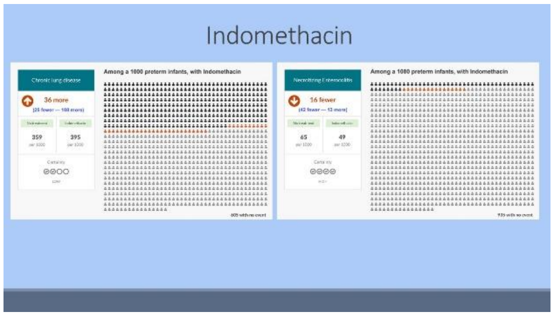

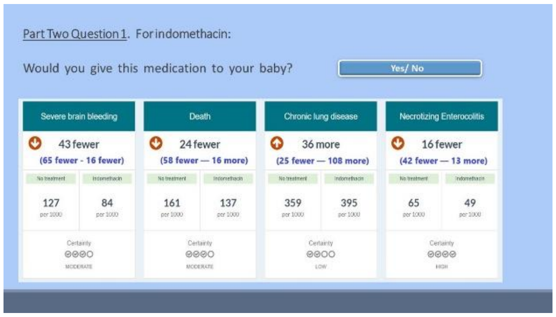

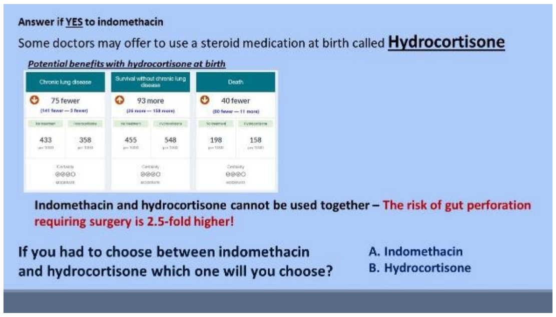

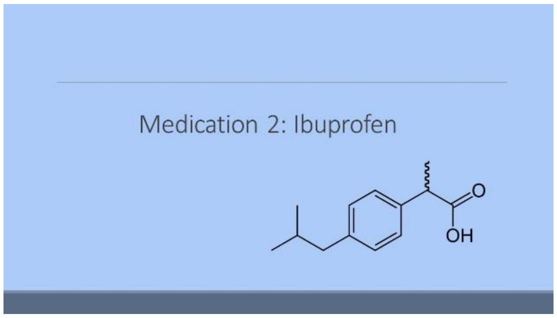

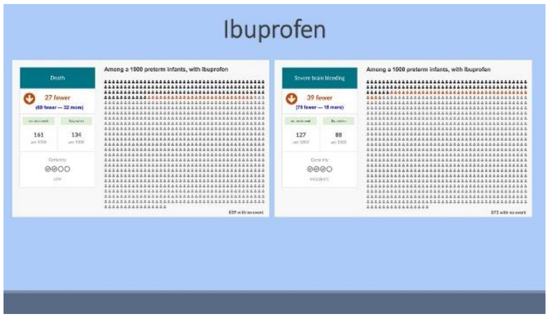

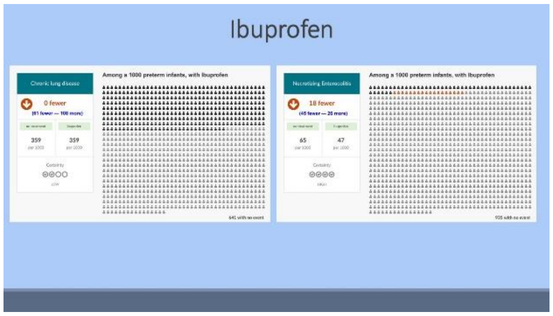

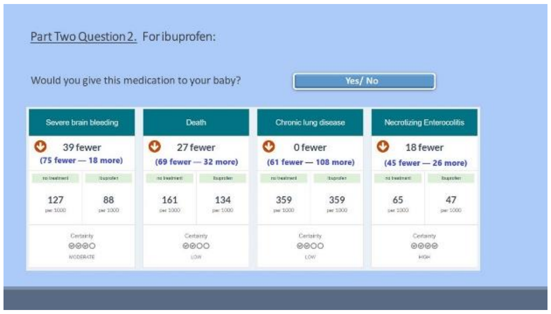

© 2023 Mitra S et al. *JAMA Network Open*.

Medication 3: Acetaminophen

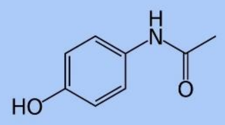

Acetaminophen

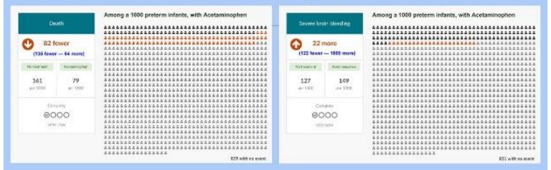

Part Two Question 3. For acetaminophen:

Would you give this medication to your baby? Yes/ No

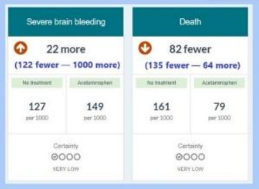

Part Two Question 4.

If given the opportunity to give one of these medications to your own baby, which would you choose?:

1. Indomethacin
2. Ibuprofen
3. Acetaminophen
4. I prefer not to give my baby any of these medications at birth

Part Three Question 1.

How important it is to have your values and preferences included in decision-making for this particular scenario (use of indomethacin/ibuprofen/acetaminophen at birth)?

1. Not important (I do not want to know the details; I will defer this decision to the doctor)
2. Somewhat important (I would like to know the benefits and harms of treatment and the rationale behind the doctor's decision; but I will follow what the doctor feels best)
3. Important (I want to have a discussion with the doctor regarding the benefits and harms related to the most important outcomes and then make a decision together)
4. Highly Important (I would like to make the decision myself based on the information provided)

Part 3 Question 2.

What were the most important factors behind your decision?

© 2023 Mitra S et al. *JAMA Network Open*.

eTable 1. Demographic Profile of Participants in the Pilot Phase 1 Study (n = 7)

Characteristic	Measure
Type of participant [n (%)]	
Parent of a very preterm infant	5 (71%)
Adult former preterm infant	2 (29%)
Age [n (%)]	
18-24	1 (14%)
25-34	1 (14%)
35-44	4 (57%)
45-54	1 (14%)
Ethnicity [n (%)]	
African descent	0 (0%)
Any visible minority	0 (0%)
Indigenous	0 (0%)
None of the above	7 (100%)
Highest level of education completed [n (%)]	
Less than high school	0 (0%)
High school	0 (0%)
College or trade school certificate or diploma	2 (29%)
University undergraduate degree	3 (43%)
University post graduate degree	2 (29%)
Country of origin [n (%)]	
Canada	6 (86%)
United Kingdom	1 (14%)

© 2023 Mitra S et al. *JAMA Network Open*.

eTable 2. Post hoc Exploratory Analysis: Responses by Participant Group

A. Value placed on outcomes

Outcome	Adult former preterm infant (n=9) [Median (IQR)]	Parent of preterm infant (n=31) [Median (IQR)]	2-sided P value (Mann-Whitney U test)
Death	100 (100-100)	100 (100-100)	Unable to compute
Severe IVH	80 (75-90)	90 (90-100)	0.08
CLD	60 (55-75)	80 (60-80)	0.15
NEC	80 (65-90)	80 (70-90)	0.47
PDA	60 (50-85)	80 (60-90)	0.25

B. Choice of pharmacoprophylaxis (when presented as the only option)

Choice of pharmacoprophylaxis	Adult former preterm infant (n=9)	Parent of preterm infant (n=31)	2-sided P value (z test)
Indomethacin	9	27	0.58
Ibuprofen	8	26	1.0
Acetaminophen	0	4	0.58

C. Choice between indomethacin and hydrocortisone

Choice between indomethacin and hydrocortisone	Adult former preterm infant (n=9)	Parent of preterm infant (n=27)	2-sided P value (Fisher's exact test)
Indomethacin	1 (11.1%)	11 (40.7)	0.22
Hydrocortisone	8 (88.9%)	16 (59.3%)	

D. Choice of pharmacoprophylaxis (when all 3 options are available)

Choice of pharmacoprophylaxis	Adult former preterm infant (n=9)	Parent of preterm infant (n=31)	2-sided P value (Fisher's exact test)
Indomethacin	5 (55.6%)	14 (45.2%)	0.43
Ibuprofen	4 (44.4%)	12 (38.7%)	
No prophylaxis	0	5 (16.1%)	

E. Importance of having participant values and preferences included in decision-making

Choice	Adult former preterm infant (n=9)	Parent of preterm infant (n=31)	2-sided P value (Fisher's exact test)
Not important	0	3 (9.7%)	0.69
Somewhat important	6 (66.7%)	16 (51.6%)	
Important	3 (33.3%)	11 (35.5%)	
Highly Important	0	1 (3.2%)	

© 2023 Mitra S et al. *JAMA Network Open*.
