# Supplementary material for: Evaluation of Health-Related Values and Preferences of Adults Who Were Preterm Infants and Parents of Preterm Infants Concerning Use of Prophylactic Cyclooxygenase Inhibitor Drugs
Source: JAMA Netw Open. 2023 Mar 9;6(3):e232273. doi: 10.1001/jamanetworkopen.2023.2273 (PMC9999240; doi:10.1001/jamanetworkopen.2023.2273)
Supplement: Supplement 2. — Data Sharing Statement [file jamanetwopen-e232273-s002.pdf]

Data Sharing Statement

Mitra. Evaluation of Health-Related Values and Preferences of Adults Who Were Preterm Infants and Parents of Preterm Infants Concerning Use of Prophylactic Cyclooxygenase Inhibitor Drugs. *JAMA Netw Open*. Published March 09, 2023.
doi:10.1001/jamanetworkopen.2023.2273

Data
Data available: Yes
Data types: Deidentified participant data
How to access data: souvik.mitra@dal.ca
When available: With publication

Supporting Documents
Document types: Informed consent form
How to access documents: souvik.mitra@dal.ca
When available: With publication

Additional Information
Who can access the data: researchers whose proposed use of the data has been approved
Types of analyses: for any purpose
Mechanisms of data availability: with a signed data access agreement
